# The Mayer Hashi Large-Scale Program to Increase Use of Long-Acting Reversible Contraceptives and Permanent Methods in Bangladesh: Explaining the Disappointing Results. An Outcome and Process Evaluation

**DOI:** 10.9745/GHSP-D-15-00313

**Published:** 2016-08-11

**Authors:** Mizanur Rahman, M Moinuddin Haider, Sian L Curtis, Peter M Lance

**Affiliations:** a University of North Carolina at Chapel Hill, Chapel Hill, NC, USA; b International Center for Diarrhoeal Disease Research, Bangladesh, Dhaka, Bangladesh

## Abstract

**Background:**

Bangladesh has achieved a low total fertility rate of 2.3. Two-thirds of currently married women of reproductive age (CMWRA) want to limit fertility, and many women achieve their desired fertility before age 30. The incidence of unintended pregnancy and pregnancy termination is high, however. Long-acting reversible contraceptives (LARCs), consisting of the intrauterine device and implant, and permanent methods (PM), including female sterilization and vasectomy, offer several advantages in this situation, but only 8% of CMWRA or 13% of method users use these methods.

**Program:**

The Mayer Hashi (MH) program (2009–2013) aimed to improve access to and the quality of LARC/PM services in 21 of the 64 districts in Bangladesh. It was grounded in the SEED (supply–enabling environment–demand) Programming Model. Supply improvements addressed provider knowledge and skills, system strengthening, and logistics. Creating an enabling environment involved holding workshops with local and community leaders, including religious leaders, to encourage them to help promote demand for LARCs and PMs and overcome cultural barriers. Demand promotion encompassed training of providers in counseling, distribution of behavior change communication materials in the community and in facilities, and community mobilization.

**Methods:**

We selected 6 MH program districts and 3 nonprogram districts to evaluate the program. We used a before–after and intervention–comparison design to measure the changes in key contraceptive behavior outcomes, and we used a difference-in-differences (DID) specification with comparison to the nonprogram districts to capture the impact of the program. In addition to the outcome evaluation, we considered intermediate indicators that measured the processes through which the interventions were expected to affect the use of LARCs and PMs.

**Results:**

The use of LARCs/PMs among CMWRA increased between 2010 and 2013 in both program (from 5.3% to 7.5%) and nonprogram (from 5.0% to 8.9%) districts, but the rate of change was higher in the nonprogram districts. Client–provider interaction and exposure to LARCs/PMs were lower in the program than nonprogram districts, and the MH program districts had higher vacancies of key providers than the nonprogram areas, both indications of a more difficult health system environment.

**Conclusion:**

The weaknesses in the health system in the MH districts apparently undermined the effectiveness of the program. More attention to system weaknesses, such as additional supportive supervision for providers, might have improved the outcome.

## Abstract (precis)

The Mayer Hashi program resulted in a modest increase in use of long-acting reversible contraceptives and permanent methods in Bangladesh, but less of an increase than in comparison nonprogram districts, which appears to have been the result of weaknesses in the health system environment in the program districts. Addressing system issues to support providers beyond training might have led to better results.

## INTRODUCTION

Long-acting and permanent methods of contraception are components of a balanced method mix. Long-acting reversible contraceptives (LARCs) comprise the intrauterine device (IUD) and implants, while permanent methods (PMs) include female sterilization and vasectomy. LARCs offer women and couples who wish to delay, space, or limit childbearing a number of advantages: they are highly effective, require little action on the part of the user, are suitable for a wide range of women, and are cost-effective over the long term.^1^^,^^2^ Discontinuation and failure rates are typically low,^3^ and LARCs have the potential to reduce unintended pregnancy and the associated risks of unsafe abortion and maternal morbidity and mortality.^2^^,^^4^ There is growing evidence that many women and couples prefer LARCs when they are available and affordable.^5^

Bangladesh has achieved a low level of fertility (total fertility rate of 2.3) and a contraceptive prevalence rate (CPR) of 62% among currently married women of reproductive age (CMWRA), increasing from about 20% in the early 1980s.^6^ Increased use of oral contraceptive pills and injectables largely accounts for the increase in CPR. Currently, only 13% of users in Bangladesh rely on LARCs and PMs compared with 74% relying on pills, injectables, and condoms.^6^ Another 13% of users rely on traditional methods. Pill use increased dramatically from 3% in the 1980s to 27% in 2014, while use of injectables increased from below 5% in the early 1990s to 12% in 2014.^6^ In contrast, prevalence of PMs increased from 7% in the early 1980s to around 10% in the early 1990s, but has declined to 6% in the 2000s. Among LARCs and PMs, tubectomy is the dominant method. IUD use is low, currently at 0.6% of CMWRA, and the current prevalence of implants is 1.7%. The number of women choosing PMs decreased substantially beginning in the early 1990s, when the number of women relying on PMs who phased out of the reproductive ages annually was larger than the number of new acceptors, which led to a decrease in prevalence.

Bangladesh has achieved a low total fertility rate of 2.3 and a contraceptive prevalence rate of 62%.

However, desire for fertility limitation has increased since the 1990s. For example, in the early 1980s, about half of mothers with 2 children wanted to have additional children.^7^ In 2011, only 20% wanted to have additional children.^8^ Among all CMWRA, 58% wanted to limit childbearing in 1993/1994, increasing to 65% in 2011,^9^ and most women achieve their desired fertility before age 30.^8^ The high reliance on short-acting and traditional methods exposes women to increased risks of method failure^10^ and early method discontinuation^11^ during the 15 to 20 years when they could become pregnant after they achieve their desired family size. A high proportion of mothers (30%) report that their last birth was unintended.^8^ Singh et al. reported there were 1.3 million menstrual regulations and abortions in 2010 in Bangladesh, yielding a pregnancy termination rate of 36 per 1,000 women ages 15–44 years old.^12^

The government of Bangladesh, along with NGOs, have encouraged efforts to increase use of LARCs and PMs to meet the needs of couples for sustained contraception. One example of these efforts is the Mayer Hashi (MH) program, funded by the United States Agency for International Development (USAID) from 2009 to 2013, to increase access to LARCs and PMs and improve the quality of services. We evaluated the impact of this program on use of LARCs and PMs and explored the pathways through which the program aimed to influence use of these methods to interpret the findings of the impact analysis.

## PROGRAM DESCRIPTION

### Family Planning Service Delivery in Bangladesh

Both the public and private sector deliver family planning services in Bangladesh. Most of the LARCs and PMs are provided by the public sector, whereas short-acting contraceptives are provided by both the public and private sector. In 2014, 92% of IUD users, 93% of implant users, 85% of vasectomy users, and 69% of female sterilization users obtained their method from the public sector. This compares with 42% of pill users, 61% of injectable users, and 15% of condom users.^6^ The Directorate General of Family Planning (DGFP) of the Ministry of Health and Family Welfare (MOHFW) is the public-sector agency that delivers both LARCs and PMs and short-acting contraceptives. Pills, condoms, and injectables are available at pharmacies, and pills and condoms are also available in convenience stores.

The DGFP delivers family planning services both in communities and health care facilities. Female family welfare assistants (FWAs) are responsible for visiting homes every 2 months to provide information and counseling and to supply pills and condoms.^13^ The family welfare visitor (FWV) provides injectables and IUDs at the family welfare center and satellite clinics in communities. In the past, only FWVs provided injectables, but FWAs have started to provide subsequent doses of injectables (the first dose is given by the FWV from a facility). There is 1 family welfare center and several satellite clinics in each union (the lowest-level administrative area) staffed by 1 FWV. The medical officer–maternal and child health (MO–MCH) provides implants, tubectomy, and no-scalpel vasectomy (NSV) from the *upazila* (subdistrict) health complex at the subdistrict headquarters. The MO–MCH or equivalent personnel also provide LARCs and PMs at other tertiary facilities throughout the country. The *upazila* family planning officer (UFPO) supervises community family planning activities. NGOs also offer family planning services at the community and facility levels in coordination with the DGFP. All service providers (FWA, FWV, and MO–MCH) receive training prior to beginning their jobs.

### The Mayer Hashi Program

The Mayer Hashi (MH) program operated in 21 of the 64 districts in Bangladesh during the period 2009–2013.^14^^-^^16^ The 21 selected districts had low use of family planning and other health care services. Seventeen of the 21 MH districts are in the eastern region of the country (Chittagong and Sylhet divisions) where people are more conservative and have more traditional beliefs compared with other regions. Family planning and other health services also tend to be weaker in this region.

The Mayer Hashi program operated in 21 of the 64 districts in Bangladesh during the period 2009–2013.

The MH interventions were grounded in the SEED (supply-enabling environment-demand) Programming Model.^17^^,^^18^ Bangladesh has well-established family planning services, and the MH interventions were added to the regular LARC/PM activities of the family planning program. The main components of the interventions are summarized in the Box.

BOX. **Mayer Hashi Interventions**

The Mayer Hashi program was grounded in the SEED Programming Model, which encompasses Supply, Enabling Environment, and Demand interventions.

**Supply Improvement**

Enhancement of knowledge and skills of providers through training and refresher training of:

- FWAs
- FWVs (3-day clinical training on providing the IUD)
- MOs–MCH (3-day clinical training on implant insertion, female sterilization, and no-scalpel vasectomy)

Expansion of services through the DGHS through training of:

- RMOs and OB/GYNs (3-day clinical training on implant insertion, female sterilization, and no-scalpel vasectomy)

System strengthening:

- Orientation of program managers (UFPOs and UHFPOs ) (1-day training on LARCs and PMs)

Logistics projection and management (also impacts the enabling environment):

- Technical assistance for program managers

Facilitation of policy formulation and policy change (also impacts the enabling environment):

- LARCs and PMs delivered through the DGHS
- LARCs and PMs available through the private sector

**Enabling Environment**

Advocacy:

- Orientation of community leaders and influential persons

**Demand Creation**

Community mobilization:

- Home visits by FWAs
- Courtyard meetings
- Cultural programs (e.g., street drama, music)
- Advocacy by local leaders and influential persons

Distribution of BCC materials (billboards, posters, and leaflets):

- In communities
- At facilities

Enhancement of provider skills:

- Training all providers in interpersonal communication

Abbreviations: BCC, behavior change communication; DGHS; Directorate General of Health Services; FWAs, family welfare assistants; FWVs, family welfare visitors; LARCs, long-acting reversible contraceptives; MOs–MCH; medical officers–maternal and child health; OB/GYNs, obstetrician/gynecologists; PMs, permanent methods; RMOs, resident medical officers; SEED, supply–enabling environment–demand; UFPO, *upazila* (subdistrict) family planning officer; UHFPO, *upazila* health and family planning officer.

### Supplies

Supply improvement includes enhancement of provider knowledge and skills, systems strengthening, and logistics management. In addition to training DGFP providers and managers, the MH program trained resident medical officers of the Directorate General of Health Services, on LARCs and PMs. The Directorate General of Health Services is an MOHFW agency that provides preventive and curative health services. The MH program trained its resident medical officers to support expansion of LARC and PM services beyond the DGFP. The program also trained obstetrician/gynecologists (OB/GYNs)—the Directorate General of Health Services personnel who provide delivery services including cesarean delivery (available in some *upazilas*)—and the program began training private providers on LARCs and PMs.

The provider training focused on enhancing clinical knowledge and skills. FWVs received a 3-day clinical training on the IUD. The training specialists assessed the theoretical knowledge and reported technical skills of FWVs on the IUD prior to the training. The training was given in 2 stages: knowledge improvement and insertion practice. The trainers used discussions to address the knowledge gaps identified in the pre-training assessment. They demonstrated IUD insertion, and then the trainees practiced among clients inserting an IUD under the observation of the trainers, who provided immediate feedback. The MOs–MCH, resident medical officers, and OB/GYNs were given a 3-day clinical training on implant insertion, tubectomy, and NSV procedures following the same model as the FWV IUD training. The FWAs who counsel couples on contraceptive methods were trained on theoretical aspects of LARCs and PMs, such as the appropriateness of each method for a particular client situation, advantages and disadvantages of LARCs and PMs, and potential side effects.

To strengthen the systems supporting LARCs and PMs, managers were provided with a day-long LARC and PM orientation. The orientation covered the importance of LARCs and PMs in the family planning service delivery system, enhancing demand for LARCs and PMs, and improving systems to better support LARC and PM use. Throughout the program, managers received help in projecting their supply needs and ensuring a secure supply of equipment and commodities for LARCs and PMs.

### Enabling Environment

Creating an enabling environment included conducting workshops with local and community leaders, including religious leaders, to encourage their participation in demand promotion for LARCs and PMs and to help overcome cultural barriers. The program published *Islam and Family Planning,* a book in Bangla, and circulated it to religious leaders. The book explains that Islam supports birth spacing and describes the available birth-spacing methods, including LARCs. At the policy level, the MH program advocated policies to support expansion of LARC and PM services through the Directorate General of Health Services and the private sector. The technical assistance provided to managers to support planning for supplies described above also aimed to contribute to strengthening the enabling environment for LARCs and PMs by improving logistics systems.

### Demand Promotion

Demand promotion encompassed training of providers in interpersonal communication, distributing behavior change communication (BCC) materials at the community and facility levels, and community mobilization. The program trained community- and facility-based providers on interpersonal communication techniques in a 1.5-day training session. The primary audience for the interpersonal communication training were the FWAs who counsel clients for LARCs and PMs at the community level, as well as other providers (FWVs, MOs–MCH). The providers were given BCC materials (flip charts and leaflets) on LARCs and PMs for client counseling. Leaflets on contraceptive methods were also produced for distribution to current and potential clients. Posters and billboards on LARCs and PMs were produced and displayed in facilities and communities.

FWAs organized community mobilization for LARCs and PMs through home visits and courtyard meetings. The primary audience for community mobilization were CMWRA. Street drama and music programs publicized LARCs and PMs, and local leaders and influential persons spoke to communities about LARCs and PMs.

The MH team routinely monitored the program by examining quarterly trends in LARC/PM acceptance in the MH intervention districts. However, there was no systematic plan for following up with providers after the training, either by the MH team or by the DGFP, to see how the training affected practice. USAID sponsored a midterm performance evaluation of the MH program that included comparing project achievements with expected results in intervention areas and interviewing stakeholders about program implementation.^15^

## METHODS: EVALUATION DESIGN

We used 2 approaches for the evaluation. To measure the impact of the MH interventions on use of LARCs and PMs, we used a before–after and intervention–comparison design. This design measures the changes in the key contraceptive behavior outcomes in the MH intervention areas relative to those in the comparison areas.^16^ We used a difference-in-differences (DID) specification to capture the impact of the program.^19^ The DID approach assumes that the change in the outcomes in the comparison group provides a good estimate of the change that would have occurred in the intervention group in the absence of the program.^20^^,^^21^ Under these assumptions, if the improvements in outcomes are significantly greater in the program areas compared with the comparison (nonprogram) areas, then we can conclude that improvements in outcomes were caused by the program.

To measure impact on use of LARCs/PMs, we used a before–after and intervention–comparison design, and a difference-in-differences (DID) specification.

To explore the pathways through which the MH interventions aimed to change contraceptive behaviors, we used an endline-only intervention–comparison design. In this design, we considered a series of intermediate indicators that measure the processes through which we expected the interventions to affect use of LARCs and PMs. This analysis is primarily descriptive.

### Data

USAID requested the impact evaluation toward the end of the MH program. Therefore, it was not possible to collect pre-program data designed for the impact evaluation. This is a common problem in evaluation in practice. To overcome this problem, we used data from the 2010 Bangladesh Maternal Mortality Survey (BMMS 2010).^22^ The BMMS 2010 was a national survey conducted during January–August 2010. Although the MH program formally began in October 2009, implementation did not begin until March 2010 and was in a sufficiently preliminary state through the end of BMMS 2010 fieldwork that it was unlikely to have an impact on population-level use of LARCs or PMs before that time.

To form program and comparison samples for the evaluation, we randomly selected 6 (of 21) program districts (Barisal, Patuakhali, Comilla, Cox’s Bazar, Moulvibazar, Sunamganj), and we selected 3 nonprogram districts (Kishoreganj, Mymensingh, Narsingdi) to match the program districts in initial LARC and PM prevalence.^16^ The nonprogram districts are from a different administrative division, but they are adjacent to some of the MH program districts, separated by a river. There is a cluster of districts in Dhaka, Chittagong, and Sylhet divisions that share a common characteristic of low performance in family planning and maternal and child health.^16^ The 3 comparison districts are in that cluster. Program districts received MH interventions to improve accessibility to and quality of LARC and PM services in addition to regular DGFP services, while nonprogram districts continued to receive regular DGFP services. This design is geared toward identification of the average effect of treatment on the treated.

The baseline data came from the BMMS 2010. The sample size in BMMS 2010 for the 6 program districts was 22,145 CMWRA, and for the 3 nonprogram districts, 9,893 CMWRA (Table 1). The endline household survey was conducted during February–May 2013 in the 9 study districts. The endline survey was a population-based household survey with a multistage sampling procedure. All CMWRA ages 13–49 who were usual residents in each selected household were eligible for the interview. The response rate for selected CMWRA was 93.4%, yielding a sample of 5,864 CMWRA (3,894 in program districts and 1,970 in nonprogram districts).

**TABLE 1.** Sample Sizes and Response Rates for the Household and Provider Surveys

Survey and Respondents	Program Districts^a^		Nonprogram Districts^b^	
	Number	Response Rate (%)	Number	Response Rate (%)
Household survey of CMWRA				
Baseline – 2010 (BMMS)	22,145	93^c^	9,893	93^c^
Endline – 2013 (MH Program)	3,894	95^d^	1,970	93^d^
Provider survey, 2013				
FWAs	118	100	62	100
FWVs	118	98	62	98
UFPOs	59	71	31	83
MOs–MCH	59	32	31	61
RMOs	53	79	28	79
OB/GYNs	53	34	28	36
*All providers*	*460*	*77*	*242*	*82*

Abbreviations: BMMS, Bangladesh Maternal Mortality Survey; CMWRA, currently married women of reproductive age; FWAs, family welfare assistants; FWVs, family welfare visitors; MH, Mayer Hashi; MOs–MCH, medical officers–maternal and child health; OB/GYNs, obstetrician/gynecologists; RMOs, resident medical officers; UFPOs, *upazila* (subdistrict) family planning officers.

a Barisal, Patuakhali, Comilla, Cox’s Bazaar, Moulvibazar, Sunamganj.

b Kishoreganj, Mymensingh, Narsingdi.

c 93% for program and nonprogram districts together.

d 94% for program and nonprogram districts together.

Additionally, the endline data collection included a survey of 702 providers in the 9 districts (460 in program districts and 242 in nonprogram districts; Table 1). The public-sector service providers of the 90 *upazilas* in the 6 program and 3 nonprogram districts were the population of interest for the provider survey. For the DGFP in an *upazila*, there is 1 MO–MCH and 1 UFPO, both of whom were included in the survey. There are 6 to 8 unions per *upazila*, and each of the unions has 1 FWV and 6 FWAs. We selected 2 unions at random per *upazila*. Within the selected union, the lone FWV and 1 randomly selected FWA were interviewed. Providers from the Directorate General of Health Services were also interviewed for each of the *upazilas*: the resident medical officer and the OB/GYN. The overall response rate of service providers was 77% in the program districts and 82% in the nonprogram districts. The response rates were low for MOs–MCH and OB/GYNs (32%–36% except for MOs–MCH in nonprogram areas, whose response rate was 61%), primarily due to high vacancy rates for these positions. The provider survey included a module on BCC products and materials available at the facilities where the providers were interviewed. Data from providers are available only at endline.

### Indicators

The outcome indicators considered in the women’s data are:

- Use of LARCs and PMs
- Use of other methods

We also considered the following program exposure indicators for women:

- Client–worker contact at home
- Client–worker contact for health care at facilities
- Client–worker contact for health and family planning care at facilities
- Acceptors of temporary methods told about PMs
- Acceptors of injectables, the IUD, and implant told about method side effects
- Acceptors of injectables, the IUD, and implant told about follow-up visits
- Women who sought care from facilities who noticed messages on LARCs/PMs
- Women who heard, saw, or read messages about tubectomy
- Women who heard, saw, or read messages about NSV
- Women who heard, saw, or read messages about the IUD
- Women who heard, saw, or read messages about implants
- Women who heard, saw, or read messages about LARCs/PMs
- Women who heard, saw, or read messages about PMs

The BMMS 2010 includes only the indicators on use of LARCs/PMs and other methods, so the DID analysis is restricted to these outcomes only. Use of LARCs/PMs is the main outcome indicator that the MH program aimed to affect. The other indicators can be compared only between intervention and comparison areas at endline.

From the provider data, we considered a number of indicators related to:

- Provider training (program exposure)
- Provider knowledge and reported practice (level of adherence to pre- and post-counseling protocols associated with LARC/PM service provision)
  ○ Pre-procedure counseling during provision of implants
  ○ Post-procedure counseling when providing IUDs
  ○ Post-procedure counseling when providing tubectomy
  ○ Information provided on method side effects

- Presence and use of BCC materials

### Analysis and Modeling

To assess and contextualize program impact, we performed both bivariate and multivariate analyses. The bivariate analysis compared the indicators of interest between program and nonprogram districts and between 2010 and 2013 (when available). The multivariable analyses involved a regression model designed to assess impact. The foundation of the impact model was a DID regression specification. We implemented this specification through a multinomial logit regression model in which the dependent variable represented the choice of the contraceptive methods by CMWRA. Three categories of choice were considered: (1) no contraception, (2) LARC or PM (IUD, implant, tubectomy, or NSV), or (3) other methods (pill, injectable, condom, or traditional methods).

To implement the DID specification to capture the potential impact of the MH program on increased use of LARCs and PMs or other methods over time, we included dummy terms for whether the district was among the 6 selected for the program as well as a dummy variable indicating whether the observation came from the baseline (BMMS 2010) or follow-up (MH endline survey). We also included an interaction term between these 2 variables, which is crucial. Finally, the specification applied to the multinomial model also included selected independent variables, such as women’s age, education, religion, household wealth quintile, and residential location (rural vs. urban).

We estimated the multinomial model in a fashion that recognizes the 3 sampling design features of the 2 surveys: stratification (both involved a stratified design), clustering, and sampling weights. To generate a unified weighting scheme between the 2 surveys, we normalized together the design selection probabilities for the observations from the 2 surveys. This, combined with the dummy variable indicating the survey (BMMS 2010 or MH endline) from which the observations were drawn, should control for any structural differences between the 2 surveys.

Because the multinomial logit is a nonlinear regression model, program impact was captured through an interaction effect obtained from the estimated model. Specifically, program impact on LARC/PM use was:

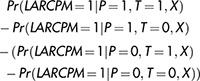


where *Pr*(*LARCPM*|*P, T, X*) is the predicted probability (from the fitted multinomial model) of using LARCs/PMs conditional on whether the individual resides in a program area (*P*=1 if residing in a program area and 0 otherwise), *T* is an indicator for whether the observation is drawn from the baseline (BMMS 2010) or endline sample, and *X* are the other characteristics (e.g., age, education) for which we controlled in the multinomial regression model. Program impact was thus the difference in predicted probabilities over time in program areas minus the same in nonprogram areas. This is in line with the classic DID approach to program impact estimation. The standard error of this program impact measure was obtained via the Delta method for functions of maximum likelihood estimates.

Beyond this causal modeling, all the indicators except for the use of LARCs/PMs and other methods were compared between program and nonprogram districts only at endline. This helped us to understand the intermediate-level variables through which the program can affect LARC/PM use.

### Additional Analysis

Bangladesh has regional variations in use of health and family planning services.^8^ Most of the MH districts (17 of 21) come from the eastern region, which tends to have weaker health systems and more conservative values. Therefore, we also examined selected indicators by region within program areas and included some health system indicators obtained from DGFP records. The additional health system indicators included vacancy level for MOs–MCH, vacancy level for UFPOs, client–provider contact, and client exposure to BCC materials. Most of these health system indicators refer to 2013, either from the endline survey or from government records. The vacancy rates of MOs–MCH and UFPOs refer to the year 2013, and we assume that the rates remained the same during 2010–2013. This assumption is plausible because the DGFP did not recruit for MO–MCH or UFPO positions during this period,^23^ and therefore no change in vacancy is expected. The vacancy level could be changed through transfer of personnel, but this is unlikely because of the way the system operates.

Most of the Mayer Hashi districts (17 of 21) were in the eastern region of Bangladesh, which tends to have weaker health systems and more conservative values.

## RESULTS

### Background Characteristics of Sampled Women

The LARC and PM use rates in program and nonprogram samples were comparable in 2010 (i.e., at baseline). We also found that these rates were comparable for program and nonprogram districts in 2004 and 2007 for the years when data were available. Women’s age, number of children, and education were comparable in program and nonprogram districts, but the nonprogram areas were more urban and had a smaller non-Muslim population than the selected program districts (Table 2). The BMMS over-sampled urban areas, while the endline MH survey did not. Estimates representative of the populations of the 9 districts considered in this study are straightforward for both surveys through the application of appropriate sampling weights.

**TABLE 2.** Background Characteristics of Sampled Women

	Program Districts		Nonprogram Districts	
	2010	2013	2010	2013
Age, years, mean	30.6	31.4	30.3	31.5
No. of children, mean	2.6	2.7	2.5	2.6
No education, %	31.1	28.5	36.7	32.0
Lowest 2 quintiles, %	39.9	42.3	40.7	35.6
Non-Muslim, %	13.7	10.4	5.7	3.5
Urban, %	33.5	25.6	44.3	27.0

Source of data: 2010 baseline data are from the Bangladesh Maternal Mortality Survey; 2013 endline data are from the Mayer Hashi endline survey.

### Provider Training

In the program districts, 63% of each group of providers, MOs–MCH, FWVs, and FWAs, reported that they received training on LARCs and PMs compared with 42% of MOs–MCH, 23% of FWVs, and 15% of FWAs in the nonprogram districts. Among OB/GYNs and resident medical officers, 28% and 5%, respectively, reported receiving training on LARCs and PMs in the program districts compared with none in nonprogram districts.

### Provider Knowledge, Skills, and Practice

Almost all the MOs–MCH and FWAs reported that they explained to clients the advantages and disadvantages of implants (Table 3). We also asked providers whether they ensured informed choice (“ensure that the client made her decision after having full information”). A higher percentage of MOs–MCH (37%) and FWAs (39%) than FWVs (14%) replied in the affirmative to this question for implants in the program districts. This percentage was lower (26% for MOs–MCHs, 23% for FWA, and 7% for FWVs) in the nonprogram districts.

**TABLE 3.** Provider Practice (%) at Endline, Mayer Hashi Provider Survey, 2013

	FWAs		FWVs		MOs–MCH	
	Program	Nonprogram	Program	Nonprogram	Program	Nonprogram
	(n = 118)	(n = 62)	(n = 116)	(n = 61)	(n = 19)	(n = 19)
Pre-counseling for implant clients						
Explain advantages and disadvantages	97	98	31	23	95	100
of implants						
Ensure informed choice	39	23	14	7	37	26
Mention probable side effects of implants						
Amenorrhea	64	81	22	20	68	100
Spotting	47	58	22	11	74	95
Post-counseling for IUD clients						
Provide the follow-up card	54	73	74	90	58	95
Determine that clients understand key	31	8	9	10	21	16
counseling points						
Post-counseling for female sterilization clients						
Provide the follow-up card	53	68	66	87	58	95
Determine that clients understand key	9	3	8	15	21	26
counseling points						

Abbreviations: FWAs, family welfare assistants; FWVs, family welfare visitors; IUD, intrauterine device; MOs–MCH, medical officers–maternal and child health.

For IUD clients, in program districts, 74% of FWVs (the only IUD provider) said they provide the follow-up card to the clients, and 54% of FWAs, who accompany the clients for the procedure and play an important role in enhancing the client–provider interaction, said they provide the follow-up card (Table 3). The follow-up card for IUD clients is an important tool for identifying method complications and their treatment, and it is expected to lead to higher continuation of the method. A higher percentage of both FWVs and FWAs reported providing the follow-up card in nonprogram districts than program districts—for FWVs, 90% vs. 74% (*P*≤.05) and for FWAs, 73% vs. 54% (*P*≤.05).

Similar patterns emerged for female sterilization clients. The level of reported knowledge and adherence of reported provider practice to standard protocols (e.g., the pre- and post-counseling protocols) was (a) generally low in both the program and nonprogram districts and (b) statistically similar in the 2 areas. However, the percentage of providing the follow-up card by FWVs was significantly greater (*P*≤.01) in nonprogram districts than program districts (Table 3). Reported practice was better among higher-level providers, such as MOs–MCH, than among FWVs and FWAs in nonprogram areas, and for some indicators in program areas.

### BCC Materials at Facilities

**FIGURE 1.**
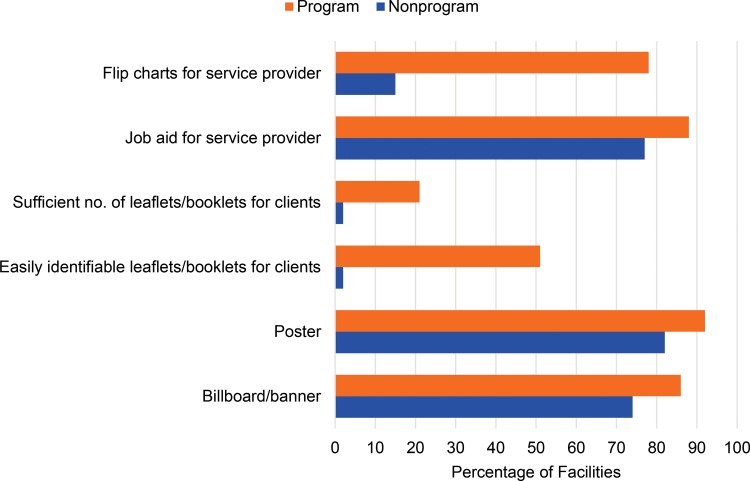
Percentage of Facilities Having BCC Materials/Products on LARCs/PMs at Endline, Mayer Hashi Provider Survey, 2013 Abbreviations: BCC, behavior change communication; LARCs, long‐acting reversible contraceptives; PMs, permanent methods.

BCC materials were commonly available in the facilities in program districts: 86% to 92% of facilities had billboards, banners, or posters on LARCs/PMs in and around the facilities (Figure 1). Such BCC materials were also commonly available in nonprogram district facilities, but less so (74% to 82% of facilities). Just over one-half of the program district facilities had a recognizable place where clients could see leaflets and booklets on LARCs/PMs, compared with only 2% in the nonprogram districts. In 88% of facilities in program districts, the providers had job aids to provide information to clients and to counsel clients on LARCs/PMs, compared with 77% in nonprogram districts (*P*≤.05).

### Client–Provider Contact

In 2013, 13% of women interviewed in the household survey reported they were visited by family planning workers in the last 3 months in program districts, compared with 23% in nonprogram districts (Table 4). The level of client–worker contact was low in both types of districts, and it is significantly lower (*P*≤.001) in the program districts than nonprogram districts. The CMWRA also had significantly higher contacts with service providers at facilities in nonprogram districts than in program districts.

**TABLE 4.** Women’s Reports of Client–Provider Contact, Informed Choice, and Exposure to Information on LARCs/PMs, Mayer Hashi Household Survey, 2013

	Percentage		Sample Size		*P* Value
	Program	Nonprogram	Program	Nonprogram	
Provider contact with CMWRA (in last 3 months)					
Visited by family planning workers	13	23	3,194	1,637	<.001
Sought health care from government facilities	30	44	3,117	1,544	<.001
Sought health or family planning care from government facilities	44	55	3,117	1,544	<.001
Informed choice – Received family planning or LARC/PM information from providers or facilities (in last 3 months)					
Temporary method acceptors who were told about PMs	40	33	410	174	NS
Injectable, IUD, and implant acceptors who were told about side effects	38	49	216	79	NS
Injectable, IUD, and implant acceptors who were reminded about follow-up visits	31	48	216	79	<.05
CMWRA who sought health or family planning care and noticed BCC materials with LARC/PM messages	42	43	1,369	854	NS
Exposure to LARC/PM Information – Heard, saw, or read messages^a^ (in last 3 months) about:					
IUDs	15	21	3,194	1,637	<.001
Implants	22	40	3,194	1,637	<.001
Tubectomy	29	35	3,194	1,637	<.001
No-scalpel vasectomy	17	13	3,194	1,637	<.001
LARCs/PMs	38	50	3,194	1,637	<.001
PMs	31	35	3,194	1,637	<.001

Abbreviations: BCC, behavior change communication; CMWRA, currently married women of reproductive age; IUD, intrauterine device; LARCs, long-acting reversible contraceptives; PMs, permanent method; NS, not significant.

a From TV, radio, newspaper/magazine, billboard/poster, folk song/theater, courtyard meeting, health/family planning worker, health facility, or friend/relative.

### Information Through Service Providers or Facilities

Quality of care of family planning services can be enhanced if contraceptive clients are told about method side effects, are reminded about follow-up visits, and are told about other method options.^24^ The percentages were less than 50% for all indictors related to these aspects of quality of care, and most of the differences between program and nonprogram districts in 2013 were not significant (Table 4). For example, only 40% and 33% of temporary method acceptors were told about permanent methods in program and nonprogram districts, respectively. Further, 38% and 49% of injectable, implant, and IUD acceptors reported they were told about method side effects in the program and nonprogram districts, respectively. Only 31% and 48% of injectable, implant and IUD acceptors were reminded about follow-up visits in program and nonprogram districts, respectively, and the difference between program and nonprogram districts was significant.

### Exposure to BCC Materials on LARCs/PMs

In the program districts, 15% of women reported that they read, heard, or saw messages on the IUD in the 3 months before the survey, 22% on implants, 29% on tubectomy, and 17% on NSV (Table 4). Women in nonprogram districts were significantly more likely to recall hearing, seeing, or reading messages on all LARCs and PMs except NSV.

### LARC/PM and Other Method Use

**FIGURE 2.**
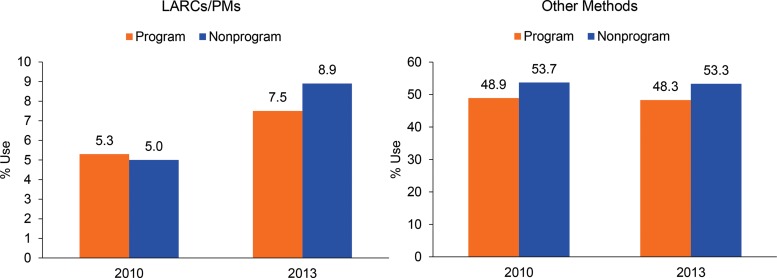
Use of LARCs/PMs and Other Methods, by Program vs. Nonprogram Districts, 2010 and 2013, Mayer Hashi Evaluation Abbreviations: LARCs, long‐acting reversible contraceptives; PMs, permanent methods.

Use of LARCs/PMs among CMWRA increased between 2010 and 2013 in both program (from 5.3% to 7.5%) and nonprogram (from 5.0% to 8.9%) districts, but the increase was significantly lower in program than nonprogram districts (Figure 2). Table 5 compares the method-specific rates between program and nonprogram districts and between 2010 (baseline) and 2013 (endline). Among the LARCs/PMs, tubectomy was the most commonly used method at baseline, used by over 3% of CMWRA in 2010. Use of other LARCs/PMs was between 0.4% and 0.8% at baseline. There was an increase in the use of each method between 2010 and 2013 in program and nonprogram districts, except for the IUD in program districts, where use declined from 0.5% to 0.4% (*P*≤.01). The increase in method use was higher in nonprogram districts than program districts, except for NSV.

Use of LARCs/PMs increased between 2010 and 2013 in both program and nonprogram districts, but the increase was significantly lower in program districts.

**TABLE 5.** Change in LARC/PM Method Use Among CMWRA Between 2010 and 2013, Mayer Hashi Program

	% Reporting Use		
	2010	2013	Change
IUD			
Program	0.5	0.4	-0.1
Nonprogram	0.4	0.6	0.2
Implant			
Program	0.7	1.2	0.5
Nonprogram	0.8	1.9	1.1
Tubectomy			
Program	3.7	4.9	1.2
Nonprogram	3.2	5.7	2.5
NSV			
Program	0.4	1.0	0.6
Nonprogram	0.5	0.8	0.3
Any LARC/PM			
Program	5.3	7.5	2.1
Nonprogram	5.0	8.9	4.0

Abbreviations: CMWRA, currently married women of reproductive age; IUD, intrauterine device; LARC, long-acting reversible contraceptive; PM, permanent method; NSV, no-scalpel vasectomy.

The CPR increased by 1.6 percentage points (from 54.2% to 55.8%) in 6 program districts and by 3.5 percentage points (from 58.7% to 62.2%) in 3 nonprogram districts. This means that most of the increase in the CPR was due to an increase in use of LARCs/PMs in both areas. Figure 2 confirms that use of other methods besides LARCs/PMs either declined or was unchanged in both program and nonprogram districts.

#### Multivariate Analysis

Table 6 shows the multinomial logit estimates of coefficients for use of LARCs/PMs and other methods. For LARCs/PMs, program impact (which, as discussed in the Methods, is captured by the interaction effect estimated from the predicted probabilities of using LARCs/PMs obtained from the multinomial logit model for each combination of program area and survey wave) is –0.017 and not significant (*P* = .14). Thus, we find that the program did not have any significant effect on increasing the probability of CMWRA using LARCs/PMs.

**TABLE 6.** Multinomial Logit Coefficient Estimates and Estimated Program (Interaction) Effect for LARC/PM Use and Other Method Use, Mayer Hashi Evaluation, 2013 (N = 37,902)

	LARCs/PMs			Other Methods		
	Coefficient	SE	*P* Value	Coefficient	SE	*P* Value
Program (ref: nonprogram)	–0.084	0.088	.34	–0.243	0.049	<.001
Year 2013 (ref: 2010)	0.616	0.157	<.001	0.145	0.070	.04
Program X Year	–0.359	0.182	.05	–0.223	0.093	.02
Age, years (ref: 25–29)						
<20	–2.396	0.280	<.001	–0.812	0.072	<.001
20–24	–1.039	0.166	<.001	–0.283	0.056	<.001
30–34	0.691	0.122	<.001	0.247	0.072	.001
35–39	1.071	0.127	<.001	0.460	0.071	<.001
40–44	0.487	0.155	.002	–0.054	0.078	.49
45–49	0.169	0.157	.28	–1.269	0.081	<.001
Education (ref: no education)						
Primary incomplete	0.242	0.110	.03	0.189	0.057	.001
Primary complete	–0.164	0.127	.20	0.273	0.053	<.001
Secondary incomplete	–0.276	0.140	.05	0.231	0.061	<.001
Secondary complete or higher	–0.589	0.197	.003	0.279	0.085	.001
Wealth quintile (ref: lowest)						
Second	–0.207	0.115	.07	0.064	0.056	.25
Middle	–0.285	0.124	.02	–0.139	0.061	.02
Fourth	–0.263	0.135	.05	–0.221	0.072	.002
Highest	–0.380	0.171	.03	–0.378	0.083	<.001
Religion (ref: Muslim)						
Non-Muslim	0.545	0.137	<.001	0.278	0.063	<.001
Sector (ref: rural)						
Urban	0.278	0.115	.02	0.286	0.054	<.001
Constant	–1.988	0.129	<.001	0.380	0.073	<.001
Program effect^a^						
Interaction effect	–0.017	0.011	.14	–0.038	0.021	.07

Abbreviations: LARC, long-acting reversible contraceptive; PM, permanent method; SE, standard error.

a Estimated from the predicted probabilities of LARC/PM use or other method use obtained from the model for each program area by survey year combination, in line with the difference-in-difference approach to estimate program impact, as described in the main body of the article.

#### Regional Variation and Health System Context

As noted previously, MH program districts were predominantly located in the eastern region of Bangladesh which tends to have weaker health systems and more conservative values. To explore the role of health system and regional context on our findings, Table 7 presents selected indicators of health system context alongside changes in the LARC/PM use observed for subregions within program and nonprogram districts. Characteristics of a stronger health system context include (1) low vacancies of MOs–MCH and UFPOs, (2) high level of client–provider contact, and (3) high level of LARC/PM information dissemination. Nonprogram districts performed better than program districts (both eastern and south-central regions) on all of these indicators. There was little difference between eastern and south-central program areas; the program areas in the eastern region had lower vacancies of UFPOs than the south-central program areas but higher vacancies for MOs–MCH, and fewer women visited health facilities for care than in the south-central program districts, which provides fewer opportunities for client–provider interactions. Therefore, it appears that the MH interventions were in districts with weaker health systems. However, the increase in use of LARCs/PMs in the south-central program districts was only slightly lower than in the nonprogram districts (located in the north-central region), while LARC/PM use lagged behind in program districts in the eastern region, suggesting that regional factors beyond the health system context may also be important.

**TABLE 7.** Indicators of Regional Health System Strength and Increase in LARC/PM Use, Mayer Hashi, 2013

	Program Districts		Nonprogram Districts
	Eastern region	South-central region	North-central region
Vacancy of UFPO, %	36	47	10
Vacancy of MO–MCH, %	56	38	19
CMWRA visited by FWA and other family planning worker,^a^ %	13	12	23
CMWRA sought health/family planning care from facilities, %	41	49	55
CMWRA heard, saw, or read about LARCs/PMs, %	38	37	50
Increase in LARC/PM use between 2010 and 2013, percentage points^b^	1.6	3.3	4.0

Abbreviations: CMWRA, currently married women of reproductive age; FWA, family welfare assistant; LARC, long-acting reversible contraceptive; MO–MCH, medical officer–maternal and child health; PM, permanent method; UFPO, *upazila* (subdistrict) family planning officer.

a Although FWAs are supposed to make home visits every 2 months, in practice less than 20% of CMWRA reported that they were visited by a family planning worker in the 6 months prior to the 2011 Demographic and Health Survey.^9^

b It was not possible to match the program and nonprogram districts in the health system characteristics that may affect couples’ LARC/PM use.

## DISCUSSION

The primary objective of this evaluation study was to estimate the impact of the Mayer Hashi program on use of LARCs and PMs in the program areas. Additionally, we explored descriptively the intermediate outcomes through which the program aimed to influence use of LARCs and PMs to interpret the findings of the impact analysis. Our findings show that the coverage of service provider training was higher in program than nonprogram districts, but higher training coverage did not necessarily translate into better provider knowledge or reported practice. Service providers in program districts were more aware of policy changes or of new policies than providers in nonprogram districts, but reported practices hardly differed between the 2 types of districts. BCC materials on LARCs and PMs were more commonly found in facilities in program districts than in nonprogram districts, but CMWRA were more likely to recall seeing, hearing, or reading messages on LARCs and PMs in nonprogram districts. The use of LARCs/PMs increased between 2010 and 2013 in both types of districts, but the rate of change was not greater in the program than the nonprogram districts.

One reason suggested for the underutilization of LARCs/PMs in many low- and middle-income countries is that these methods are more challenging for the health system to deliver than short-acting methods.^2^^,^^5^ Our analysis suggests that the Mayer Hashi program districts were programmatically less “ready” than nonprogram districts to provide LARCs and PMs. Notably, program districts had a higher rate of vacancy of MOs–MCH, the only provider of implants and female and male sterilization, than nonprogram districts. Also, vacancy of UFPOs, the *upazila* (subdistrict) family planning manager who supervises family planning outreach activities, was higher in the program than nonprogram districts.

The lack of impact of the Mayer Hashi interventions on LARC/PM use at the population level could be related to structural constraints associated with program/service readiness.

The government monitoring and supervision system is also weak. The ongoing monitoring and supervision of the trained providers was the responsibility of the government of Bangladesh and not in the scope of the Mayer Hashi program, which resulted in weaknesses in support for translation of knowledge and skills acquired in training into behavior change. The providers did not receive any mentoring or supportive supervision, a crucial element of performance improvement. Moreover, LARC/PM service delivery requires carefully designed activities to deal with quality of care, clients’ conservative outlook in the program districts, and many other challenges. The program districts, with poor program readiness and other problems, had an increase in LARC/PM use of 2 percentage points, compared with 4 percentage points in the nonprogram districts. This increase of 2 percentage points is considerable in view of all the challenges in the program districts. CMWRA in program districts reported less contact with the health system than women in nonprogram districts. Family planning workers’ home visits and visits by women to heath facilities for family planning or other care were both higher in the nonprogram districts.

Because of the lower desire for fertility limitation and the weaker health systems in the eastern region of Bangladesh, it may be more appropriate to focus on increasing use of short-acting methods in the immediate future.

Some interventions were undertaken at the policy level to influence the enabling environment. In particular, policy-level work was successful in expanding provision of LARCs and PMs to include the Directorate General of Health Services and private/NGO providers, and the project began training these providers too. These larger system changes will likely take time to affect the health system environment substantially, however.

Because of the generally lower desire for fertility limitation and weaker health systems in the eastern region where the Mayer Hashi districts were largely located, it may be more appropriate to focus on increasing use of short-acting methods in the immediate future. In contrast, the western region has an environment that is likely to be more conducive to LARC/PM promotion because of the higher desire for fertility limitation and higher use of contraception there, and the region’s stronger family planning program infrastructure.

The MH team routinely examined quarterly trends in LARC/PM acceptance in the MH districts. These analyses of routine service statistics indicated that LARC/PM use was increasing and that the project was exceeding its objectives.^15^ The lack of impact of the project becomes apparent when the increase in LARC/PM use in program districts is compared with the increase in nonprogram districts. Such an analysis is beyond the scope of most project monitoring plans, but this illustrates the limitations of relying primarily on monitoring service statistics in program areas to track progress toward objectives.

There is a relatively long series of intermediate steps from the project interventions to the outcome of increased LARC/PM use at the population level. The midterm evaluation commissioned by USAID identified some limitations in intermediate steps (e.g., BCC activities) from key informant interviews and site visits. The evaluation noted that providers who were trained in postpartum IUD insertion had little opportunity to apply those skills because postpartum women were not strongly motivated to accept IUDs.^15^ Such implementation insights are helpful. However, as noted previously, there was no systematic plan for following up with providers to assess the effect of training on practice, or for monitoring other intermediate steps in the program. Increased attention to process evaluation to complement outcome monitoring and impact evaluation can identify interventions that are effective and those that are ineffective, and inform corrections to the program.^25^

### Limitations

This article reports on an evaluation of a program operating at scale under real-world conditions. Such evaluations face a number of design and implementation challenges that require pragmatic and creative approaches.^26^^,^^27^ We faced a common challenge: the evaluation was requested toward the end of the project, so we could not collect baseline data specific to the evaluation. For example, it was not possible to ensure that the program and nonprogram districts had similar health system capacities.

We did not have baseline data on provider knowledge and behavior or on women’s knowledge of and attitudes toward LARCs and PMs. Therefore, our analysis of the intermediate outcomes through which the program aimed to influence use of LARCs and PMs is limited to a comparison between program and nonprogram districts at endline only and is therefore more descriptive. It is possible that provider knowledge and practice were weaker in program than in nonprogram districts at baseline, and that the interventions brought the providers in the program districts up to the level of those in nonprogram districts. However, the baseline use of LARCs/PMs was the same in program and nonprogram areas, so it does not appear that any baseline differences in provider knowledge and practice were associated with a difference in use of LARCs/PMs. Therefore, while we might be missing some effects of training on the intermediate step of provider knowledge and practice, it is unlikely that this limitation explains the lack of effect of the interventions on LARC/PM use.

The DID estimation strategy to evaluate the program effect on LARC/PM use was the strongest evaluation design available to us. The DID model rests on the assumption that the change observed in the nonprogram districts is a proxy for the change that would have been observed in the program districts in the absence of the interventions. It is not possible to test this assumption directly, although trends in LARC/PM use appear to have been similar in program and nonprogram districts between 2004 and 2010. This is a limitation of DID analyses in general, however.

## CONCLUSION AND RECOMMENDATIONS

Use of LARCs and PMs is increasing slowly in Bangladesh, but the increase is greater in districts or regions where the health system is stronger or the desire for family limitation is stronger. Provider vacancy is universally a crucial factor in efforts to improve health services. The availability of appropriate, high-quality providers for LARCs and PMs has been a challenge in Bangladesh for many years in spite of its substantial progress in health care.^23^^,^^28^

An immediate solution to these challenges is unlikely; therefore, Bangladesh should seek alternative and/or supplemental avenues to provide high-quality LARC/PM services. The privatization of LARC and PM services is one option. The Bangladesh government has opened up LARC/PM services to the private sector recently, and combined efforts of the public-, private-, and NGO sectors should be encouraged, especially in view of the recent growth of the private sector for family planning and other reproductive and child health services.^6^^,^^29^ Further operations research is needed to discover innovative, affordable, and effective interventions to improve LARC/PM service delivery within a constrained health system, and more attention to process evaluation will improve our understanding of implementation and progress along the program pathway to complement outcome monitoring and impact evaluations.

## Notes

**Peer Reviewed**
